# Homologous Use of Allogeneic Umbilical Cord Tissue to Reduce Knee Pain and Improve Knee Function

**DOI:** 10.3390/life12020260

**Published:** 2022-02-09

**Authors:** Ruben Berrocal Timmons, Kiminobu Sugaya, Lori Deneke Bane

**Affiliations:** 1 Regenerative Medicine and Pain Management Physicians, 3406 Santa Rosa Drive, Gulf Breeze, FL 32563, USA; lori@advancedregenmed.com; 2 Burnett School of Biomedical Sciences, College of Medicine, University of Central Florida, 6900 Lake Nona Blvd, Orlando, FL 32827, USA; ksugaya@ucf.edu

**Keywords:** umbilical cord tissue, UCT, knee pain, VAS, WOMAC

## Abstract

To determine if knee pain subjects who received cryopreserved umbilical cord tissue (UCT) injected into knee joints experience less knee pain, better function, decreased physical limitations, and reduction of medications (opiates, NSAIDs, and acetaminophen) over a 24 week period, Visual Analog Scale (VAS), Western Ontario and McMaster Universities Osteoarthritis Index (WOMAC), and medication usage data were recorded for 30 consenting human knee pain subjects receiving UCT at a single site in the United States. Subject profile information was gathered and analyzed to gain insight into the effects of age, sex, and BMI on improvement over time. Mean resting VAS scores and mean VAS scores with activity improved over 24 weeks (from 1.95 to 0.83 and from 6.28 to 2.87, respectively, *p* < 0.001). There was no strong evidence of a correlation between sex and VAS scores. There were statistically significant correlations for BMI vs. pre-injection VAS with activity scores and Age vs. pre-injection VAS with activity scores (r = 0.402, *p* = 0.028 and r = 0.434, *p* = 0.017, respectively). Mean WOMAC scores improved from 44.7 to 18.5 over 24 weeks (*p* < 0.001). 77.8% of patients who used medications at the beginning of the study reduced or eliminated medication use. The analysis demonstrates that injections with UCT decrease pain, improve physical function, and allow for less medication use for at least 24 weeks.

## 1. Introduction

Osteoarthritis (OA), the most common type of disease affecting joints, is one of the most predominant causes of chronic pain and disability in developed nations, including the United States. Specifically, knee OA represents over 80% of the types of osteoarthritis, affecting almost one-fifth of American adults over the age of 45 years [1]. Almost 23% of U.S. adults (an estimated 54.4 million people) report having doctor-diagnosed arthritis. Even without a doctor’s diagnosis, almost half of American adults experience limitations in performing activities of daily living, secondary to arthritis [2]. In recent decades, high body mass index (BMI) has become rampant in the United States. Being overweight or obese is a risk factor for knee OA due to joint overloading and adiposity-induced inflammation [1]. Based on an analysis of data from 2010–2012 from the National Health Interview Survey (NHIS), it is projected that 26% of adults will be diagnosed with arthritis by 2040. Arthritis and other rheumatic conditions are leading causes of work disability among U.S. adults [2]. Adults with arthritis are almost three times more likely to fall and suffer injury than their non-arthritic counterparts [3]. In a group of patients with knee and hip OA, 25% were unable to perform major activities of daily living, and 40% described their health as fair to poor, which ranks them high in disability-adjusted life years [4]. Because knee pain limits physical activities such as walking, bending, and climbing stairs, reducing pain can improve physical activity and conditioning [5]. 

Senescing joint tissues accumulate more cartilage loss due to inflammation, oxidative stress, and matrix metalloproteases (MMP) activation [6]. Joint replacement surgeries are most often due to OA [7]. Considering both direct and indirect costs associated with OA, average annual costs from 2008–2014 totaled USD 486.4 billion. In 2013, the OA treatment costs were USD 140,300 [8].

Current treatments for joint pain are limited, and include medical management, injection therapy, and surgery. Pain medications are linked with significant morbidity and social concern. Over the course of the year 2019, 10.1 million people over the age of 12 years misused opioids, the overwhelming majority of which were prescription pain relievers. Of those, approximately 1.6 million people over the age of 12 years were diagnosed with an opioid use disorder [9]. Physicians struggle with the increasing responsibility and cost of prescribing opiates for chronic pain as well as heightened scrutiny monitoring patient usage and preventing abuse. Almost 500,000 deaths were attributed to opioid overdose, including prescription and illicit opioids, from 1999–2019 [10].

Non-steroidal anti-inflammatory drugs (NSAIDs) are often the first treatment tried by patients suffering from chronic pain. Hospital admission rates due to upper and lower gastrointestinal events secondary to NSAID and aspirin drug use were significant with a mortality rate of almost 6% of admitted patients. About 30% of these deaths were attributed to the use of low-dose aspirin [11]. While most common NSAIDs have a minimal toxic effect, NSAID use and overdoses continue to increase, as reported by poison control centers nationwide. Adverse drug interactions, especially in vulnerable patients, increase morbidity and mortality [12]. Specifically, drug–drug interactions result in liver injury and high incidences of hospitalizations [13]. 

While steroid injections provide short-term relief of pain, side effects such as diabetes exacerbation, weight gain, cataracts, osteoporosis, difficulty sleeping, menstrual changes, and increased risk of infection should be considered [14]. Disease-modifying anti-rheumatic drugs (DMARDs) may also increase the risk of infection and can cause liver or kidney damage. Some patients do not tolerate DMARDs, and they may not be safe for those who are or are trying to become pregnant [15].

Another possible injectate, viscosupplementation, often requires multiple injections of hyaluronate. Patients experience relief for only 8–12 weeks, if at all [16]. These types of injections offer a cushion with no type of medicinal effects and are costly. 

Finally, surgery for joint replacement associated with chronic pain is very costly and may have a limited effect on pain and disability [7,8]. Given the high cost, limitations, and side effects associated with traditional methods of treatment of chronic joint pain, alternative approaches to relief are needed. 

Another option is umbilical cord tissue (UCT) which is available for clinical use from registered global tissue banks. Methods of preparation and forms of umbilical cord tissue allografts may vary, affecting the specific contents and clinical results. 

UCT from healthy live births contains regenerative, anti-inflammatory, immunomodulatory, and wound-healing properties due to the many growth factors and cytokines present in UCT at a higher concentration than in other biologics. These may play a factor in their inflammation-reducing, pain-reducing, and musculoskeletal-healing properties [17,18]. UCT contains prostaglandin E_2_ (PGE2), interleukin-10, vascular endothelial growth factor, and tissue inhibitors of metalloproteinases that suppress cartilage damage. These proteins exhibit powerful anti-inflammatory and anti-fibrotic effects in osteoarthritic joints [19,20]. PGE2 “reprograms” macrophages from the M1 phenotype (inflammatory) to the M2 phenotype (anti-inflammatory) [21]. UCT stimulates various metabolic processes such as general protein and collagen synthesis, which in turn reduce pain. UCT is safe in humans and animals and its uses include burn treatment, painful chronic wound healing, and in the shoulder, foot, and ankle surgeries [22,23,24,25].

## 2. Materials and Methods

This study was conducted under an Advarra IRB-approved protocol (Pro00040024), and we obtained informed consent from all patients.

Placental and umbilical cord tissue donated by volunteers, screened and free of viral and bacterial disease, undergoing caesarian section was processed to obtain UCT. The UCT was minimally manipulated under aseptic conditions and cryopreserved with dimethyl sulfoxide (DMSO) 10% solution, retaining a large amount of its initial matrix microstructure and cytokine profile. The samples were sent off to an outside laboratory to confirm the sterility of the product and freedom from endotoxins. The product was then shipped to the end user on dry ice and then transferred to a −80 °C freezer until use. The cryopreserved UCT was used homologously as a cushion or protective barrier of membranous tissue placed in or over damaged joint tissue in patients with joint pain due to osteoarthritis. Previous conventional medical management, such as pharmacological and physical therapy, failed for the patients receiving UCT for joint pain, making it medically necessary to proceed with interventional treatment. 

After patient education and consent, the human patient underwent an ultrasound-guided lateral suprapatellar injection utilizing 1 mL of thawed cryopreserved UCT along with 4 mL sterile normal saline under direct ultrasound visualization after the knee was prepped with alcohol. The product was thawed to room temperature and was then diluted to a volume wherein the amount of DMSO injectate was 2.5%. The needle utilized was a 22 gauge 1-½ inch needle. The post-procedure evaluation involved alertness, pain, stable vital signs, and unchanged neurologic status after 15 min. The patient was discharged in stable condition with postoperative instructions. 

While UCT knee injections are a daily practice at this clinic, outcomes have not been adequately reported. The fact that these patients failed conservative measures was a prerequisite to imply a clinical control. The pre-condition was used as a control. 

**Figure 1:**
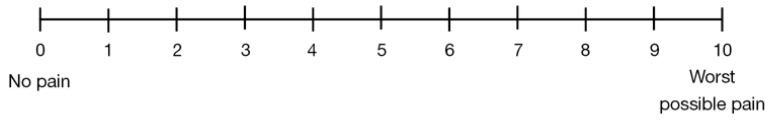
Visual Analogue Scale. Used by patients to relate pain level at rest and with activity.

**Figure 2:**
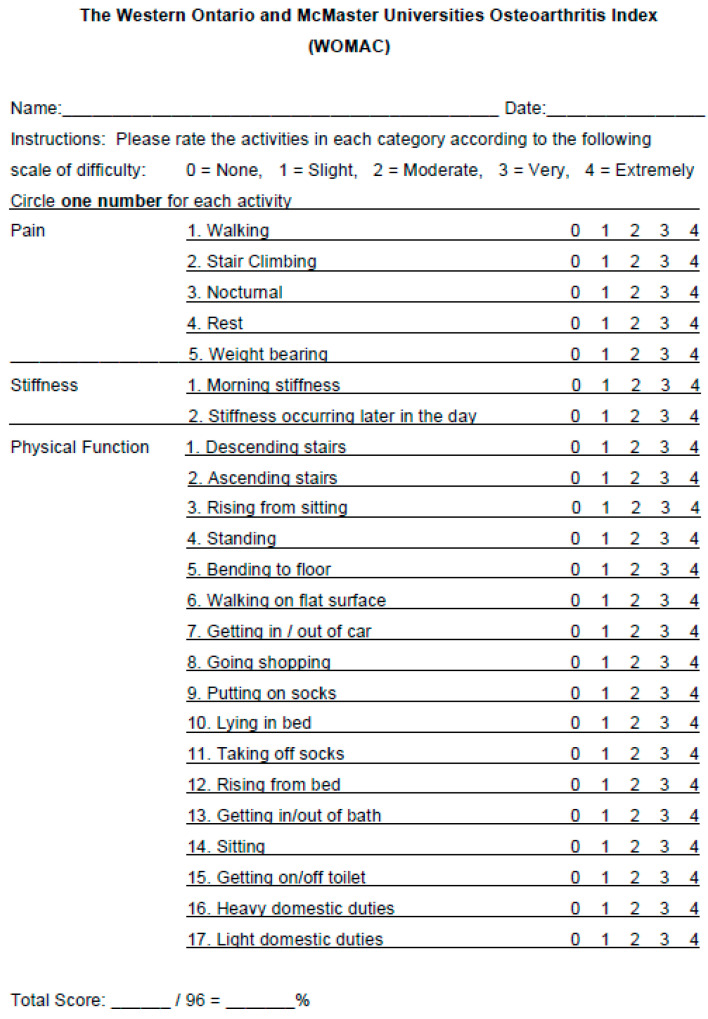
WOMAC English version 3.0 was used by patients to relate pain, stiffness, and physical function. Scores range from 0–96.

To report outcomes after UCT injection for joint pain, medical charts of 30 consenting adult subjects with knee joint pain were reviewed for Visual Analogue Scale (VAS) pain scores (Figure 1), Western Ontario and McMaster Universities Osteoarthritis Index (WOMAC) daily activity function English version 3.0 (Figure 2), opiate usage, NSAID usage, and serious adverse events [26]. The patients had failed conservative treatments (including but not limited to hyaluronic acid and steroid injections) and were treated with UCT at a single institution. Patients were included in the study regardless of diabetes, hypertension, thyroid disease, or other comorbidities. Patients continued their current medication, exercise, and dietary regimen. 

The pain was assessed by the patient using the VAS before the UCT injection as a baseline and after 1 h, 24 h, 1 week, 2 weeks, 8 weeks, 12 weeks, and 24 weeks of the UCT injection for both resting scenarios and with activity scenarios. Pain levels were subjective complaints utilizing the Visual Analogue Scale from 1–10. Patients rated their pain as per the VAS scale at rest and while engaging in normal activities of daily living. These were further supported using the WOMAC. Pain, stiffness, and physical function were assessed using the WOMAC questionnaire before the UCT injection as a baseline, and 2, 8, 12, and 24 weeks after the UCT injection. The use of opiates, NSAIDs, and other medications was recorded before the UCT injection as a baseline and 24 h, 1 week, 2 weeks, 8 weeks, 12 weeks, and 24 weeks after the UCT injection.

All the data were analyzed by analysis of variance (ANOVA) followed by post hoc analysis, which differed depending on the data set being analyzed (Appendix A). 

## 3. Results

Medical records for 30 patients presented substantially comprehensive data regarding demographics and outcomes. The mean age was 63.0 ± 10.9 years with a range of 36–84. Table 1 shows the details of patient age versus sex. 

There was no statistical difference found between the sexes and VAS scores (resting or with activity) at the 24-week review, based on independent sample *t*-tests. However, a statistically significant correlation was found between age and the pre-injection VAS (resting) score (r = 0.434, *p* = 0.017), showing higher pre-injection VAS (resting) scores for older patients. 

Two-thirds of the patients received treatment for their right knees, and one-third received treatment for their left knees. Table 2 shows the details of patient gender versus injection (treatment) site. 

The patient population’s body mass index (BMI) was 29.2 ± 7.0 with a range of 18.0–50.2 (Table 3). 

A statistically significant correlation was found between BMI and the pre-injection VAS (active) score (r = 0.402, *p* = 0.028), showing a higher pre-injection VAS (with activity) score for patients with a higher BMI.

Mean VAS scores (resting) improved from 1.95 to 0.83 over 24 weeks (*p* < 0.001), while mean VAS scores (with activity) improved from 6.28 to 2.87 (*p* < 0.001) for the same period.

ANOVA tests followed by Tukey’s HSD post hoc analysis were conducted for VAS at rest scores over the 24-week period and VAS with activity scores over the 24-week period. The results from those analyses revealed that mean VAS scores with activity were statistically different (higher) than the mean VAS scores at rest for every time interval.

### 3.1. VAS Scores with Activity

- Mean VAS scores with activity at baseline were statistically different (higher) than the mean VAS scores with activity for every post-injection time interval;
- The *p*-value for the ANOVA for VAS scores with activity was 1.18 × 10^−15^;
- Overall, there was a decrease in the mean for VAS with activity scores after the injection.

### 3.2. VAS Scores at Rest

- Mean VAS scores at rest were statistically different (higher) than the mean VAS scores at rest for every time interval;
- The *p*-value for the ANOVA for VAS scores at rest was 0.000561;
- Overall, there was a decrease in the mean for VAS at rest scores after the injection.

**Figure 3:**
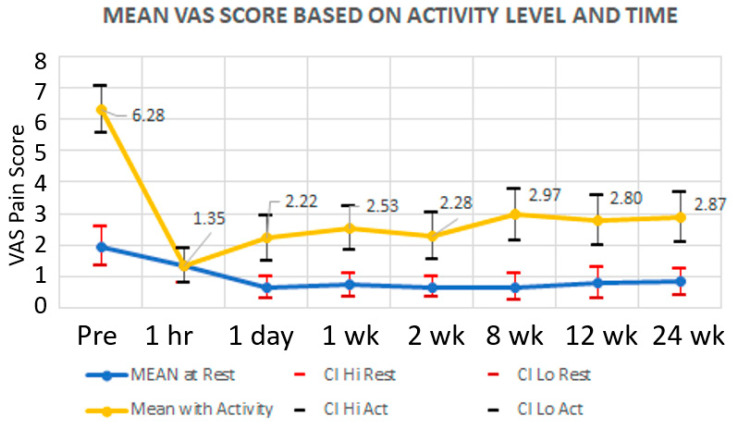
Mean VAS Scores (resting and with activity) over time with standard deviation. We did not record 1 h evaluation with activity; at rest VAS score was used for comparison.

Graphical representation of the mean VAS scores (at rest and with activity) over time with standard deviation is shown in Figure 3. 

**Figure 4:**
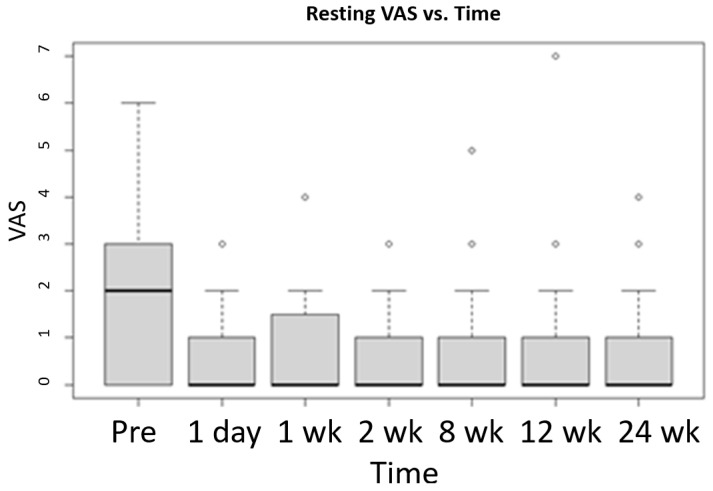
Mack-Wolfe test results for VAS (Resting) scores vs. time since injection with standard deviation. The median, quartiles, and outliers are indicated. Data: x and g, Ap* = −3.0623, *p*-value = 0.008, alternative hypothesis: theta_1 ≤ … ≤ theta_p ≥ … ≥ theta_k, *p* = 5 (the fifth bar graph).

**Figure 5:**
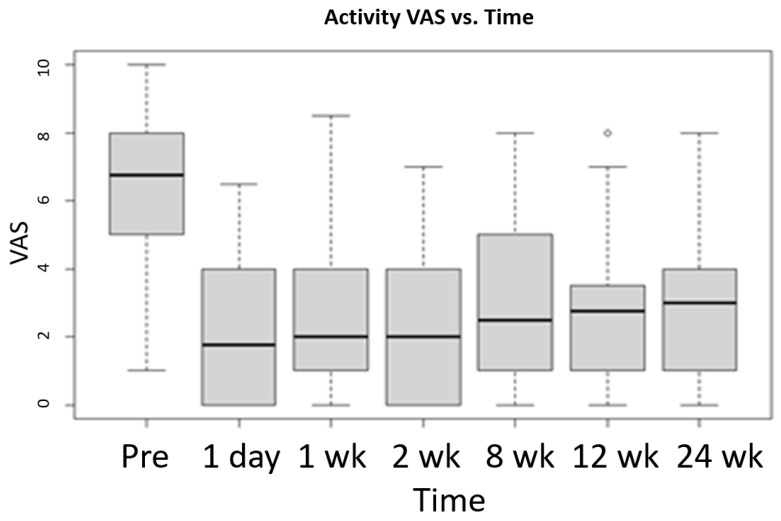
Mack-Wolfe test results for VAS (with activity) scores vs. time since injection with standard deviation. The median, quartiles, and outliers are indicated. Data: x and g, Ap* = 1.9614, *p*-value = 6 × 10^−4^, alternative hypothesis: theta_1 ≤ … ≤ theta_p ≥ … ≥ theta_k, *p* = 4 (the fourth bar graph).

The Mack-Wolfe test is a non-parametric technique used to test for a U or umbrella shape in the median (i.e., a peak or valley). It works even when the curve is very non-symmetrical about the valley (i.e., when a quadrative regression would not fit well). Execution of the Mack-Wolfe test on the VAS scores revealed that there was strong evidence of a valley in the VAS (resting) score median at 8 weeks and a valley in the VAS (with activity) score median at 2 weeks. See Figure 4 and Figure 5 for graphical representation of the Mack-Wolfe test results for the VAS scores at rest and with activity, respectively.

**Figure 6:**
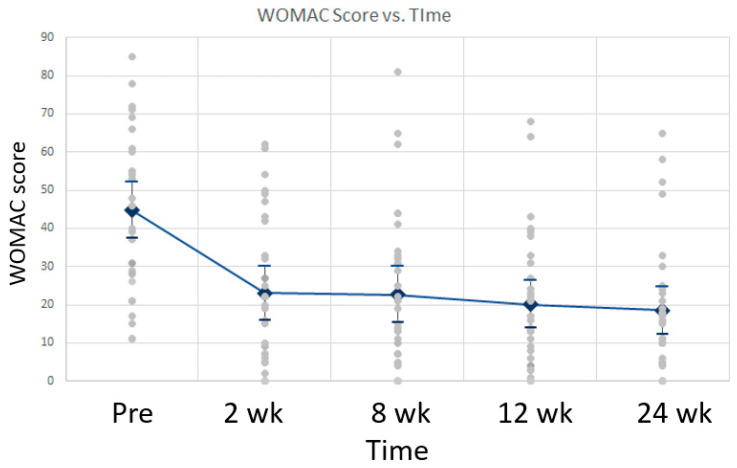
Mean WOMAC scores over time with standard deviation. WOMAC scores decreased over time compared to baseline, indicating less pain, stiffness, and improved physical function following treatment.

WOMAC-measured physical function scores improved from 44.7 to 18.5 over the 24-week review period (*p* < 0.001). ANOVA for WOMAC scores over the 24-week review period revealed that the mean pre-injection WOMAC score was statistically different from the mean WOMAC scores at 2 weeks, 8 weeks, 12 weeks, and 24 weeks. This is supported by a *p*-value of 1.34 × 10^−6^. Otherwise, the mean WOMAC scores at 2 weeks, 8 weeks, 12 weeks, and 24 weeks were not statistically different from each other. Graphical representation of the mean WOMAC scores over time is shown in Figure 6.

Plotting VAS scores over time revealed a slow linear increase with much uncertainty (*p* = 0.1972), suggesting further data need to be collected over a longer period to draw a strong conclusion; however, this linear model suggests it would take an estimated 1162 days (3 years, 8 weeks) to return to pre-injection VAS with activity scores without further intervention. (Table 4). 

The factor coding was coded. The sum of squares was Type III Partial. The model F-value of 1.71 implies the model was not significant relative to the noise. There was a 19.27% chance that this large F-value could occur due to noise.

*p*-values less than 0.0500 indicate significant model terms. There were no significant model terms in this case. Values greater than 0.1000 indicate the model terms are not significant. When there are many insignificant model terms (not counting those required to support hierarchy), model reduction may improve the model.

The lack of fit F-value of 0.38 implied that the lack of fit was not significant relative to the pure error. There was an 82.49% chance that a lack of fit F-value this large is due to noise. Non-significant lack of fit is good, as we wanted to model the fit. 

A quadratic model was tried but it did not improve the model fit. The model is described below:

(1) Activity VAS=2.416+0.003326 (Days Post Injection)

Given that the average pre-VAS score was 6.28333, we estimated that it would take about 1162 days post-injection (3 years and 8 weeks) for VAS scores to return to pre-Injection levels, on average.

(2) $$\mathrm{Days}\;\mathrm{to}\;\mathrm{Return}\;\mathrm{to}\;\mathrm{Pre}\text{-}\mathrm{Injection}\;\mathrm{Scores}=\frac{\;\mathrm{Activity}\;\mathrm{VAS}-2.416}{0.003326}=\frac{\;6.28333-2.416}{0.003326}=1162\;\mathrm{Days}$$

**Figure 7:**
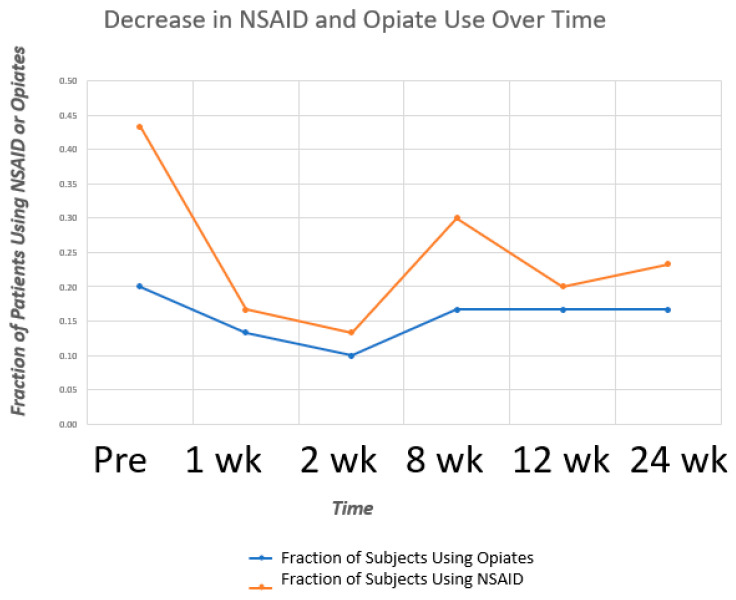
NSAID and opiate use at baseline and after treatment. Patient medication use decreased over time compared to baseline.

Overall, of the patients who used medications at the beginning of the study (18), 77.8% of them reduced or eliminated medication use, as shown in Figure 7. 

Analysis of medication use by patients over the study period revealed:

- 30% (n = 9) of the patients studied never took medication throughout the course of the study period;
- 13.3% (n = 4) of the patients studied continued to take medications throughout the entire course of the study period;
- 20% (n = 6) of the patients studied went from daily medication use to no medication use by the end of the study period;
- 13.3% (n = 4) of the patients studied reduced their medication frequency from daily to occasional by the end of the study period;
- 13.3% (n = 4) of the patients studied reduced their medication frequency from occasional to none by the end of the study period;
- One person went from using no medications to using CBD oil on the knee at the end of the study period. Although that patient had decreasing WOMAC and VAS at rest scores throughout the study period, their VAS with activity score did not change significantly throughout the study period (VAS range: 3–4);
- One person went from using no medications to occasionally using acetaminophen at the end of the study. This patient’s VAS and WOMAC scores remained consistent throughout the study;
- One person went from using no medications to using acetaminophen or ibuprofen daily. This person’s VAS and WOMAC scores initially decreased after the injection, but increased back up over time to pre-injection scores;
- The number of patients using opioids decreased by 16.7% from pre-injection to 24-week follow-ups, and the number of patients using NSAID decreased by 46.1% from pre-injection to 24 weeks follow.

Medication data were binarized at 24 weeks (any medicine vs. none). A Welch two-sample *t*-test showed strong evidence of a difference for VAS with activity scores and those using medications; that is, subjects using medications reported higher VAS active scores (*p*-value = 0.064).

No serious adverse events were reported throughout 24 weeks. Between 8 and 12 weeks, one patient fell and reinjured the knee and was reinjected at 5 months. The late intervention was without complication. All three outcomes represent a consistent result with significant improvement over time. Extended follow-up is expected at 52 weeks for all patients. 

## 4. Discussion

A chart review of 30 patients with joint pain showed the clinical benefits of injecting UCT for knee pain. Improvements in pain and physical function and a reduction in medication use (opiates and NSAIDs) began promptly after treatment. It was sustained over at least a 2-week period, extending to 24 weeks in most cases. UCT offers an important alternative to opiate use, a national CDC epidemic due to dependency, overdose, and abuse, with related difficulty managing patients on opiates [9,10].

In many instances, patients were physically limited and deconditioned secondary to their pain. Counseling on weight reduction and a transition to physical activity is a key adjunctive measure in achieving improvement in normal activities. Prior to treatment, many patients exhibited increased BMI and were physically deconditioned. This is a factor that may complicate or hinder recovery and functional restoration. Therefore, patients must be counseled on the reduction in BMI for sustained outcomes. 

This study demonstrated that the injections were effective for 24 weeks; however, we have continued to follow these patients over the course of 2 years. We have observed that many patients experience more than 24 months of relief compared to baseline with just one injection. This is consistent with the extrapolation projection, as demonstrated in our ANOVA linear model (Table 4). 

While a single steroid injection is less expensive than a single UCT injection, a patient may receive up to four steroid injections each year, increasing the morbidity associated with each injection. This is compared to UCT, which requires only one injection and has not been associated with any adverse events or increased morbidity and may provide several years of relief. 

While this review supports safety and efficacy, its limitation is that it is not a double-blinded randomized study. Additional studies must confirm the data substantiating pain relief and improved function in patients with arthritic joint pain before acquiescing to joint replacement.

In conclusion, UCT injections reduce pain, physical incapacity, and medication use (specifically opiates and NSAIDs) in patients experiencing knee pain for at least 24 weeks. As a result, this would allow patients to postpone or delay joint replacement until BMI and physical condition is optimized.

## Figures and Tables

**Table 1:** Patient age vs. sex summary.

Age (Years)	Overall	Female	Male
Sample Size	30	19	11
Mean Age	63	64.2	61
Min Age	36	36	49
Max Age	84	84	78
Std. Dev	10.94	12.10	8.75

**Table 2:** Injection site vs. sex summary.

Injection Site	Overall	Female	Male
Sample Size	30	19	11
Right Knee	20	12	8
Left Knee	10	7	3

**Table 3:** Patient BMI vs. sex summary.

BMI	Overall	Female	Male
Sample Size	30	19	11
Mean BMI	29.2	26.9	33.1
Min BMI	18.0	18.0	23.8
Max BMI	50.2	39.3	50.2
Std. Dev.	6.97	5.54	7.71

**Table 4:** ANOVA for the linear model for activity VAS.

Source	Sum of Squares	df	Mean Square	F-Value	*p*-Value	
Model	7.86	1	7.86	1.71	0.1927	Not significant
A-Time (Days)	7.86	1	7.86	1.71	0.1927	
Residual	818.42	178	4.60			
Lack of Fit	7.03	4	1.76	0.3770	0.8249	Not significant
Pure Error	811.38	174	4.66			
Cor Total	826.28	179				

**Figure A1:**
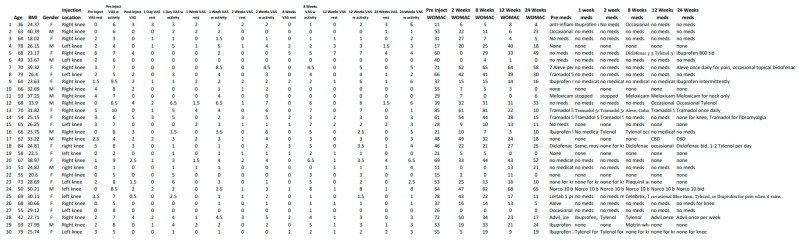
Raw data for baseline VAS at rest and with activity, follow up VAS at rest and with activity, baseline WOMAC, follow up WOMAC, and medication usage over time.

## Data Availability

Data can be found in Appendix A.

## References

[1] Wallace I.J., Worthington S., Felson D.T., Jurmain R.D., Wren K.T., Maijanen H., Woods R.J., Lieberman D.E. (2017). Knee osteoarthritis has doubled in prevalence since the mid-20th century. Proc. Natl. Acad. Sci. USA.

[2] Arthritis-Related Disabilities and Limitations. www.cdc.gov/arthritis/data_statistics/disabilities-limitations.htm.

[3] Arthritis-Related Statistics. www.cdc.gov/arthritis/data_statistics/arthritis-related-stats.htm.

[4] Michaud C.M., McKenna M.T., Begg S., Tomijima N., Majmudar M., Bulzacchelli M.T., Ebrahim S.E., Ezzati M., Salomon J.A., Kreiser J.G. (2006). The burden of disease and injury in the United States 1996. Popul. Health Metr..

[5] Fukutani N., Iijima H., Aoyama T., Yamamoto Y., Hiraoka M., Miyanobu K., Jinnouchi M., Kaneda E., Tsuboyama T., Matsuda S. (2016). Knee Pain during Activities of Daily Living and Its Relationship with Physical Activity in Patients with Early and Severe Knee Osteoarthritis. Clinical Rheumatology.

[6] Goldring M.B., Otero M. (2011). Inflammation in osteoarthritis. Curr. Opin. Rheumatol..

[7] Murphy L., Helmick C.G. (2012). The impact of osteoarthritis in the United States: A population-health perspective. Am. J. Nurs..

[8] Hochberg M.C., Cisternas M.G., Watkins-Castillo S.I. Osteoarthritis. BMUS: The Burden of Musculoskeletal Diseases in the United States. www.boneandjointburden.org/fourth-edition/iiib10/osteoarthritis.

[9] Find Help: ATOD. SAMHSA. https://www.samhsa.gov/find-help/atod.

[10] Understanding the Epidemic. www.cdc.gov/drugoverdose/epidemic/index.html.

[11] Lanas A., Perez-Aisa M.A., Feu F., Ponce J., Saperas E., Santolaria S., Rodrigo L., Balanzo J., Bajador E., Almela P. (2005). A nationwide study of mortality associated with hospital admission due to severe gastrointestinal events and those associated with nonsteroidal antiinflammatory drug use. Am. J. Gastroenterol..

[12] Weigard T.J., Vernetti C.M. (2020). Nonsteroidal Anti-inflammatory Drug (NSAID) Toxicity. http://emedicine.medscape.com/article/816117-overview.

[13] Datta A., Flynn N.R., Barnette D.A., Woeltje K.F., Miller G.P., Swamidass S.J. (2021). Machine Learning Liver-Injuring Drug Interactions with Non-Steroidal Anti-Inflammatory Drugs (NSAIDs) from a retrospective electronic health record (EHR) cohort. PLoS Comput. Biol..

[14] Epidural Corticosteroid Injections Johns Hopkins Medicine.

[15] RA Medications: What Are Disease Modifying Antirheumatic Drugs?. https://www.rheumatoidarthritis.org/treatment/medications/dmards/.

[16] Viscosupplementation for Knee Osteoarthritis Cleveland Clinic. https://my.clevelandclinic.org/health/articles/14982-viscosupplementation-for-osteoarthritis-of-the-knee.

[17] Arutyunyan I., Fatkhudinov T., Sukhikh G. (2018). Umbilical cord tissue cryopreservation: A short review. Stem Cell Res. Ther..

[18] Davies J.E., Walker J.T., Keating A. (2017). Concise review: Wharton’s jelly: The rich, but enigmatic, source of mesenchymal stromal cells. Stem Cells Transl. Med..

[19] Gupta A., El-Amin S.F., Levy H.J., Sze-Tu R., Ibim S.E., Mafulli N. (2020). Umbilical cord-derived Wharton’s jelly for regenerative medicine applications. J. Orthop. Surg. Res..

[20] Fernandes J.C., Martel-Pelletier J., Pelletier J.P. (2002). The role of cytokines in osteoarthritis pathophysiology. Biorheology.

[21] Frisbie D.D., Ghivizzani S.C., Robbins P.D., Evans C.H., McIlwraith C.W. (2002). Treatment of experimental equine osteoarthritis by in vivo delivery of the equine interleukin-1 receptor antagonist gene. Gene Ther..

[22] Heusinkveld M., de Vos van Steenwijk P.J., Goedemans R., Ramwadhdoebe T.H., Gorter A., Welters M.J.P., van Hall T., van der Burg S.H. (2011). M2 macrophages induced by prostaglandin E2 and Il-6 from cervical carcinoma are switched to activated M1 macrophages by CD4^+^ Th1 cells. J. Immunol..

[23] Acevedo P. (2020). Successful treatment of painful chronic wounds with amniotic and umbilical cord tissue: A case series. SAGE Open Medical Case Reports.

[24] Ackley J.F., Kolosky M., Gurin D., Hampton R., Masin R., Krahe D. (2019). Cryopreserved amniotic membrane and umbilical cord particulate matrix for partial rotator cuff tears: A case series. Medicine.

[25] DeMill S.L., Granata J.D., McAlister J.E., Berlet G.C., Hyer C.F. (2014). Safety analysis of cryopreserved amniotic membrane/umbilical cord tissue in foot and ankle surgery: A consecutive case series of 124 patients. Surg. Technol. Int..

[26] Woolacott N.F., Corbett M.S., Rice S.J.C. (2012). The use and reporting of WOMAC in the assessment of the benefit of physical therapies for the pain of osteoarthritis of the knee: Findings from a systematic review of clinical trials. Rheumatology.

